# FUS driven circCNOT6L biogenesis in mouse and human spermatozoa supports zygote development

**DOI:** 10.1007/s00018-021-04054-8

**Published:** 2021-12-22

**Authors:** Teresa Chioccarelli, Geppino Falco, Donato Cappetta, Antonella De Angelis, Luca Roberto, Martina Addeo, Marco Ragusa, Davide Barbagallo, Liberato Berrino, Michele Purrello, Concetta Ambrosino, Gilda Cobellis, Riccardo Pierantoni, Rosanna Chianese, Francesco Manfrevola

**Affiliations:** 1 Dipartimento di Medicina Sperimentale, Sez. Bottazzi, Università degli Studi della Campania “L. Vanvitelli”, Via Costantinopoli 16, 80138 Napoli, Italy; 2 Dipartimento di Biologia, Università di Napoli “Federico II”, Napoli, Italy; 3 Istituto di Ricerche Genetiche Gaetano Salvatore, Biogem scarl, Ariano Irpino, Avellino, Italy; 4 Dipartimento di Scienze Biomediche e Biotecnologiche, Università di Catania, Via Santa Sofia 97, 95123 Catania, Italy; 5 Dipartimento di Scienze e Tecnologie, Università del Sannio, Benevento, Italy

**Keywords:** Backsplicing, Cannabinoid receptor I, circRNAs, Sperm maturation, Embryo development

## Abstract

Circular RNA (circRNA) biogenesis requires a backsplicing reaction, promoted by inverted repeats in *cis*-flanking sequences and *trans* factors, such as RNA-binding proteins (RBPs). Among these, FUS plays a key role. During spermatogenesis and sperm maturation along the epididymis such a molecular mechanism has been poorly explored. With this in mind, we chose circCNOT6L as a study case and wild-type (WT) as well as cannabinoid receptor type-1 knock-out (*Cb1*^*−/−*^) male mice as animal models to analyze backsplicing mechanisms. Our results suggest that spermatozoa (SPZ) have an endogenous skill to circularize mRNAs, choosing FUS as modulator of backsplicing and under CB1 stimulation. A physical interaction between FUS and CNOT6L as well as a cooperation among FUS, RNA Polymerase II (RNApol2) and Quaking (QKI) take place in SPZ. Finally, to gain insight into FUS involvement in circCNOT6L biogenesis, FUS expression was reduced through RNA interference approach. Paternal transmission of FUS and CNOT6L to oocytes during fertilization was then assessed by using murine unfertilized oocytes (NF), one-cell zygotes (F) and murine oocytes undergoing parthenogenetic activation (PA) to exclude a maternal contribution. The role of circCNOT6L as an active regulator of zygote transition toward the 2-cell-like state was suggested using the Embryonic Stem Cell (ESC) system. Intriguingly, human SPZ exactly mirror murine SPZ.

## Introduction

Mammalian cells retain thousands of circular RNAs (circRNAs) with tissue-specific and cell type expression patterns [1–4].

The molecular mechanism promoting circRNA biogenesis requires a backsplicing reaction that—unlike canonical splicing—covalently binds a downstream splice donor site reversely with an upstream splice acceptor site [5]. The circularization of exonic circRNAs is promoted by inverted repeats in *cis*-flanking sequences [6], as well as by *trans* factors, such as RNA-binding proteins (RBPs) which have conserved binding sites in the flanking introns [7, 8]. The *Fused protein in Sarcoma* (*FUS*) gene plays a crucial role in circRNA biogenesis during Embryonic Stem Cell (ESC) differentiation [9]. Other RBPs have also been implicated [4, 10, 11]. Conversely, Adenosine (A) to Inosine (I) editing by ADAR deaminase, proposed to destabilize the base-pairing necessary for backsplicing reaction, perturbs circRNA biogenesis [12]. *N*^6^-methyladenosine (m^6^A) has recently been suggested to promote circRNA accumulation in late spermiogenesis to compensate for massive degradation of linear mRNAs, with several aspects of the involved molecular machinery to be unveiled [13].

In this scenario, circRNA biogenesis and degradation during spermatogenesis and sperm maturation along the epididymis have been poorly explored. To this scope, we used circCNOT6L as a study case considering that it has been recently discovered in human spermatozoa (SPZ), localized in sperm head, with a potential role in embryo development [3]. Its linear counterpart (CNOT6L-mRNA) is a key component of the eukaryotic de-adenylase complex CCR4-NOT [14], implicated in the maternal mRNA decay pathway, during the oocyte-to-embryo transition [15]. Although genetic deletion of *Cnot6L* yields viable mice, females in the offspring are severely sub-fertile [16]: eggs develop slower and, frequently, arrest at prometaphase [17]. In addition, the over-translation of maternal undegraded mRNAs causes microtubule-chromosome disorganization that leads to a spindle assembly checkpoint and meiotic cell cycle arrest [16].

The biogenesis of circCNOT6L as well as FUS and ADAR levels in testis and sperm were explored in cannabinoid receptor type-1 (CB1) knock-out (*Cb1*^*−/−*^) male mice, chosen for their altered reproductive phenotype in comparison to wild-type (WT) animals [18–23]. Our results led us to hypothesize CB1 involvement in backsplicing that we confirmed by in vitro assays.

Focusing on sperm cells, we evaluated their ability to circularize mRNAs during their transit along the epididymis, choosing FUS protein as a key modulator of backsplicing under CB1 stimulation. Therefore FUS protein localization was assessed by immunofluorescence; then the physical interaction between FUS and CNOT6L and the cooperation among FUS, RNA Polymerase II (RNApol2) and Quaking (QKI) were demonstrated by RNA immunoprecipitation assay (RIP) and immunoprecipitation assay (IP), respectively. Finally, to gain insight into FUS involvement in circCNOT6L biogenesis, FUS expression was reduced through an RNA interference approach.

Encouraged by a bioinformatic prediction according to which several downstream mRNA targets of circCNOT6L are associated with embryo development, we evaluated the paternal transmission of FUS and CNOT6L to oocytes, during fertilization, verified the only involvement of sperm in such a mechanism using parthenogenetically activated oocytes and defined the role of circCNOT6L during the early phases of zygote development using a 2-cell-like ESC system.

Intriguingly, what was observed in mouse SPZ was also confirmed in humans.

## Material and methods

### Experimental animals

WT male mice or males carrying a *Cb1* null mutation [24] were used in this study. Heterozygous mice were bred on a CD1 background (Charles River Laboratory, Lecco, Italy) before generating male mice (WT, *Cb1*^*−/−*^). Adult males (4–8 months) under anesthesia were sacrificed by cardiac perfusion with PBS (pH 7.6) to clean peripheral tissues (testes, epididymides and SPZ) from blood contaminants. In detail, testes were rapidly removed and stored at − 80 °C, while epididymides were dissected and used to collect total epididymal SPZ or SPZ from *caput* (*caput* SPZ) and *cauda* (*cauda* SPZ) regions, and relative epididymal tissues (SPZ-deprived epididymis), depending on the experimental procedure, as described below.

### Human sperm collection

Human semen samples were obtained from normozoospermic volunteer donors (*n* = 5) through masturbation after 3–5 days of sexual abstinence and collected in sterile sample containers, which were delivered to the laboratory within 1 h after ejaculation. The sperm samples were allowed to liquefy for 30 min at 37 °C and were then purified on a discontinuous density gradient. In particular, using a 40% and 80% discontinuous PureCeption (Cooper Surgical, Trumbull, CT, United States) gradient, we were able to purify viable and motile SPZ from the base of the 80% PureCeption fraction (“A-SPZ” of good quality) and abnormal SPZ from 40% PureCeption fraction (“B-SPZ” of poor quality). An aliquot of all sperm samples was used to assess sperm vitality by Trypan blue staining (Trypan Blue, 0.4% Solution, 17-942E Lonza). The analysis of live SPZ was performed under a light microscope by counting the percentage of live/total SPZ.

A- and B-SPZ fractions were then treated with Somatic Cell Lysis Buffer (SCLB) consisting of 0.1% SDS, 0.5% Triton X-100 in DEPC-H_2_O, to eliminate possible contamination by somatic cells. In brief, sperm pellets were incubated with an appropriate volume of SCLB on ice for 30 min. After lysis, SPZ were centrifuged at 300×*g* for 15 min at 4 °C and then washed twice with sperm washing medium (HTF-IrvineScientific^®^). Following SCLB treatment and microscope examination carried out to verify the elimination of somatic cells, an aliquot of A- and B-SPZ samples was used to re-evaluate the number of live SPZ under a light microscope to exclude effects on sperm vitality and concentration induced by the technical procedure (data not shown). A- and B-SPZ pellets were stored at − 80 °C for RNA or protein extraction and dried on slides and finally stored at − 20 °C for immunofluorescence analysis.

###  Sperm collection from mouse *caput* and *cauda* epididymis

Total epididymis (from *n* = 3 WT and *n* = 3 *Cb1*^*−/−*^) or *caput/cauda* epididymis (from *n* = 3 WT) were separately immersed in PBS (pH 7.6) and cut to let SPZ flow out from the ducts. Samples of total and/or *caput* and *cauda* SPZ were then filtered throughout cheesecloth to eliminate fragments of epididymal tissue and centrifuged at 1500×*g* for 30 min at 4 °C. The epididymal fragments were separately frozen as pieces of total or *caput* and *cauda* epididymis deprived of sperm cells.

After centrifugation, the SPZ pellet was incubated on ice for 30 min with SCLB (0.1% SDS, 0.5% Triton X-100 in DEPC-H_2_O) to eliminate possible contamination by somatic cells. After lysis, SPZ were centrifuged at 800×*g* for 15 min at 4 °C and then washed twice with PBS. Aliquots of total epididymal SPZ from WT and *Cb1*^*−/−*^ or *caput* and *cauda* SPZ from WT mice were both stored at − 80 °C for RNA or protein extraction and dried on slides to be finally stored at − 20 °C for immunofluorescence analysis.

### ACEA in vitro treatment of mouse testis and SPZ, and human SPZ

ACEA (arachidonyl-2-chloroethylamide), a selective CB1 receptor agonist, was obtained from Sigma-Aldrich (A9719; Milan, Italy). The drug was dissolved in dimethylsulfoxide (DMSO) according to the manufacturer’s instructions.

WT testes with a feeble notch in tunica albuginea (*n* = 3 for experimental group) were incubated in PBS (6 ml) for 90 min at room temperature (RT), with vehicle (0.005% DMSO; control group, CTRL) or with ACEA at 0.1 μM, 1 μM and 10 μM. After treatment the testes were kept at − 80 °C.

C*aput* SPZ from WT mice or human B-SPZ fraction from normozoospermic volunteers were purified as described above and incubated in PBS (1 ml) for 30 min at 37 °C with vehicle (0.005% DMSO; CTRL) or with ACEA at 1 μM. After treatment SPZ were centrifuged at 1500×*g* for 20 min at 4 °C and washed twice with PBS. The sperm pellet was stored at -80 °C.

### Immunofluorescence analysis on mouse and human SPZ

Mouse and human SPZ dried on slides as above reported were fixed in 4% paraformaldehyde (sc-281692; Santa Cruz Biotechnology, Heidelberg, Germany) for 20 min at RT and then permeabilized with 0.1% Triton X-100 (X100; Sigma-Aldrich, Milano, Italy). After permeabilization, blocking was conducted with 10% of donkey serum (ab7475; Abcam, Cambridge, UK) for 30 min at RT and the cells were then incubated with anti-FUS antibody (Ab) (PA5-52610; Invitrogen, Milano, Italy), overnight at 4 °C. Following three washes in Dulbecco’s PBS (DPBS, 1X), a fluorescein isothiocyanate (FITC) conjugated Ab was used (711-095-152; Jackson ImmunoResearch, Cambridge, UK) for 1 h at 37 °C. Nuclei were labeled with DAPI (D9542; Sigma-Aldrich, Milano, Italy) and slides were analyzed with a Zeiss LSM700 confocal microscope.

### Vesicle shuttle in vitro experiment

*Caput* and *cauda* epididymis (from *n* = 8 WT) were separately pulled in PBS (pH 7.6) and cut to let SPZ flow out from the ducts. *Caput* and *cauda* SPZ samples were then filtered throughout cheesecloth to eliminate fragments of epididymal tissue and centrifuged at 1500×*g* for 30 min at 4 °C. The resulting fluid was further clarified via centrifugation (16,000×*g* for 30 min at 4 °C) with the supernatant yielding the Epididymal Luminal Fluid (ELF) [25]. ELF purity was checked for possible sperm contaminations under a light microscope (Leica Microsystems Inc., Milano, Italy). The SPZ pellet was obtained as described above; both the SPZ pellet and ELF were used for in vitro treatments as follows.

For each experimental group, 10 × 10^6^ washed SPZ from *caput* epididymis were incubated for 30 min at 37 °C in 1 ml of: (1) PBS (CTRL group); (2) *Caput* ELF; (3) *Cauda* ELF; (4) *Cauda* ELF pre-treated for 2 h at 37 °C with anti-CD9 Ab (sc-13118; Santa Cruz Biotechnologies, Heidelberg, Germany) at concentration of 10–1000 ng/ml [26]; (5) *Cauda* ELF in presence of ACEA 1 μM.

After treatment SPZ were centrifuged at 1500×*g* for 20 min at 4 °C and washed twice with PBS. The sperm pellet was kept at − 80 °C.

### Mouse Zygote Manipulation

Murine one-cell zygotes (F) were obtained as reported by Ragusa et al. [27]. In brief, for mature oocyte collection, natural cycling CD1 females (*n* = 12), in the estrus phase, were mated with fertile (*n* = 6) or vasectomized/sterile (*n* = 6) CD1 males, following the scheme: 1 male with 1 female. Pregnant female mice at 0.5 day* post coituum* (*dpc*) were euthanized by cervical dislocation and the uterus was explanted. Then, an uterine horn was laid in a drop of Hyaluronidase from the bovine testes (H4272; Sigma-Aldrich, Milano, Italy) in M16 medium (M7292; Sigma-Aldrich, Milano, Italy) 1 mg/ml, and the fertilization ampoule was broken to release embryos and the cells surrounding them. Then, oocytes were collected in a drop of PBS to wash them from blood residues. The F obtained from CD1 females mated with fertile males, were selected by stereomicroscope observation, excluding not fertilized oocytes.

With the aim of collecting mature not fertilized oocytes (NF), we induced pseudopregnancy in females, by mating them with vasectomized/sterile males, to allow them to behave hormonally as pregnant [28]. With this approach, we selected by stereomicroscope observation the NF—in metaphase II (MII) stage—that extruded the first polar body (PB), comparably to F zygotes.

Six pools (1 pool/female), each containing 10 F or 10 NF, were used for expression analysis. For RNA and protein extraction, each experimental oocyte pool (F and NF) was subjected to thermolysis as described by Di Pietro et al. [29]: the samples were resuspended in 10 μl of RNase-free water and incubated for 1 min at 100 °C, put in ice for 1 min and, finally, vortexed for 30 s. Released RNA and proteins were analyzed by qRT-PCR and western blot, respectively, as described below.

### Parthenogenetic activation of murine oocytes

Parthenogenetic activation of murine oocytes (PA) was performed as previously reported by Kaufman [30]. Briefly, CD1 female mice (*n* = 6) were injected with 5 International Units (IU) of pregnant mare serum gonadotropin (PMSG) and 5 IU of human chorionic gonadotropin (hCG). After 16–18 h, female mice were sacrificed. The oocytes, surrounded by cumulus cells, were recovered and then released into freshly prepared 7% ethanol in DPBS solution, in a 3 cm sterile tissue culture dish, for 5 min at RT. After three washes with DPBS and two washes with M2 medium (M7167**,** Merck, Germany), the cumulus masses were transferred to single drops of M16 medium (MR-016-D, Merck, Germany), covered by paraffin oil and incubated at 37 °C for 5 h. Cumulus cells were then removed by Hyaluronidase (final concentration of 0.5–1 mg/mL), and the activated oocytes were classified under a phase contrast microscope. Finally, the PA—in metaphase II (MII) stage—that extruded the PB, comparably to NF and F zygotes, were transferred to single drops of M16 medium under paraffin oil and stored.

### ESC culture and Retinoic Acid treatment

Generation of E14tg2^pcDNA3_prZScan4_LNGFR^ was previously described [31]. The cells were cultured on gelatin-coated dishes in ES complete medium: GMEM (Sigma-Aldrich, Milano, Italy) supplemented with 15% FBS (GE Healthcare, Milano, Italy), l-glutamine 2 mM (Gibco, Dublin, Ireland), sodium pyruvate 1 mM (Gibco, Dublin, Ireland), MEM amino acids 1X (Gibco, Dublin, Ireland), penicillin/streptomycin 100 U/ml (Gibco, Dublin, Ireland), 2(*β*)-mercaptoethanol 0.1 mM (Gibco, Dublin, Ireland), LIF 1000 U/ml (Millipore, Burlington, United States), and Geneticin^(^™^)^ 137.5 μg/ml (Gibco, Dublin, Ireland). The medium was changed daily and cells were routinely split every 2–3 days and incubated at 37 °C in 5% CO_2._ For Retinoic Acid (RA) treatment, the cells were trypsinized and plated on gelatin-coated dishes in ES complete regular medium (RM) or ES complete medium supplemented with 1.5 μM all-*trans*-RA (Sigma-Aldrich, Milano, Italy) (RA). After 72 h, the cells were washed with ice-cold PBS (1X), trypsinized into a single cell suspension, and incubated with MACSelect^(^™^)^ LNGFR MicroBeads (Miltenyi Biotec, Bologna, Italy) in PBS (1X) supplemented with 5 mM EDTA, and 0.5% BSA (PBE Buffer) at 4 °C for 20 min. Magnetically labeled cells were then isolated using the AutoMACS Pro Separator (Miltenyi Biotec, Bologna, Italy) with the “posseld2” program and prZscan4_LNGFR positive and negative fractions from RM and RA treatment homogeneously collected, according to the manufacturer’s protocol.

### RNA interference

For small interfering RNA (siRNA)-mediated knockdown of *Fus* mRNA, E14tg2^pcDNA3_prZScan4_LNGFR^ stable cell line was thawed and cultured for 3 days in RM medium. Subsequently, the cells were reverse transfected with 5, 10, 25 and 50 nM of *Fus* siGENOME SMART pool siRNA or siGENOME Non-Targeting as scramble control (siScr) (Dharmacon, Inc., United States). Briefly, siFus and siScr, at the selected concentrations, were complexed with DharmaFECT1 transfection reagent, according to the manufacturer’s procedures. Then siRNA:DharmaFECT complex was added to 12-well plates and incubated for 30 min at RT. Finally, 1.4 × 10^5^ cells were plated in each well containing the selected siRNA concentration, in antibiotic-free cell culture medium. The cells were harvested after 48 or 72 h from siRNA transfection and used for total RNA or cell lysates preparation, as reported below.

### Total RNA preparation

Total RNA was extracted from murine tissues, murine sperm cells, human sperm cells and murine E14tg2^pcDNA3_prZScan4_LNGFR^ cells transfected with *Fus* siRNA or siScr, using Trizol Reagent (Invitrogen Life Technologies, Paisley, UK) following the manufacturer’s instructions. In brief, samples were homogenized in Trizol Reagent (1 ml Trizol Reagent/mg tissue or 5–10 × 10^6^ sperm cells); after homogenization, samples were incubated for 5 min at 20 °C to allow the complete dissociation of nucleoprotein complexes. Then 0.2 ml chloroform/ml Trizol Reagent was added and the sample centrifuged at 12,000×*g* for 15 min at 4 °C. The aqueous phase was transferred to a fresh tube and total RNA was precipitated by mixing with isopropyl alcohol (0.5 ml/ml Trizol Reagent) and 1 µl glycogen (20 mg/ml) to promote the precipitation of small size RNAs. After centrifugation at 12,000×*g* for 10 min at 4 °C, the RNA pellet was washed with 75% ethanol, centrifuged at 7500×*g* for 10 min at 4 °C and dissolved in an appropriate volume of DEPC-treated water. The quantity (ng/µl) and purity (260/280 and 260/230 ratios) of total RNAs were assessed with a NanoDrop 2000 spectrophotometer (Thermo, Waltham, MA, United States). To remove potential contamination of genomic DNA, RNA aliquots (10 µg) were treated with 2U DNase I (RNase-free DNase I, Ambion, Thermo Fisher Scientific, Massachusetts, United States). The RNAs were then preserved at − 80 °C until the next step.

### RNA expression analysis by One-Step Evagreen qRT-PCR

We investigated the expression of circCNOT6L and its linear counterpart (CNOT6L-mRNA) through One-Step Evagreen qRT-PCR reaction using a kit containing qRT-PCR enzyme mix and an Evagreen qPCR Mastermix (Applied Biological Materials Inc., Ferndale, WA, United States), according to the manufacturer’s instructions. All reactions were performed using 50 ng of total RNA on a CFX-96 Real Time PCR System (Biorad, Milano, Italy). Assays were carried out in triplicates and included a melting curve analysis for which all samples displayed single peaks for each primer pairs. A negative control, without RNA, was also included. RNA expression was evaluated through CFX Manager software (Biorad, Milano, Italy). Normalization was performed using *Actin* or *GAPDH* (glyceraldehyde 3-phosphate dehydrogenase) as housekeeping genes, for mouse and human samples, respectively. Normalized fold expression (nfe) of circCNOT6L and CNOT6L mRNA was calculated by applying the 2^−∆∆Ct^ method. Results were expressed as mean value of nfe ± SEM.

### PCR primer design

Specific primers for circCNOT6L and CNOT6L-mRNA were designed through the online tool Primer-BLAST (http://www.ncbi.nlm.nih.gov/tools/primer-blast/). To make primers specific for the circular isoforms, we designed primers spanning the backsplicing junctions. We also designed specific primers for the housekeeping genes used for normalization: *Actin* or *GAPDH*, for mouse and human samples, respectively. All primer sequences are shown in Table 1.

**Table 1:** Primers sequence and annealing temperatures

Gene primers	Sequences *5′–3′*	Tm (°C)
*Mmu-circCNOT6L S*	ATTTACGGGTGTTGCCTTATGA	56
*Mmu-circCNOT6L AS*	TGCGAGGATCTGGAGGATCA	
*Mmu-CNOT6L-mRNA S*	CGTCCTGGGCATTAAACTGG	56
*Mmu-CNOT6L-mRNA AS*	CCACGATCCTTCAATGCTGG	
*Mmu-circACTB S*	GGCTGTATTCCCCTCCATCG	55
*Mmu-circACTB AS*	CCAGTTGGTAACAATGCCATGT	
*Mmu-FUS S*	GGTGGTGGAGGCAACTATGG	56
*Mmu-FUS AS*	GTCACTTCCGCCCATGCCGC	
*Hsa-circCNOT6L S*	GCCTTATGAACTTGGTCGGCT	56
*Hsa-circCNOT6L AS*	TTCTGCGAGGATCTGGAGGAT	
*Hsa- CNOT6L-mRNA S*	TCGCAGTTCATCCAGAGCAG	54
*Hsa- CNOT6L-mRNA AS*	ACGGCAGAATTTGGTCTCGT	
*Hsa-circGAPDH S*	TGCACCACCAACTGCTTAGC	58
*Hsa-circGAPDH AS*	GGCATGGACTGTGGTCATGAG	

### RNA binding protein immunoprecipitation assay (RIP)

For RIP assay, 1 × 10^7^ of mice *caput* SPZ or human B-SPZ were lysed in 500 µl of RIP lysis buffer (50 mM Tris–HCl pH 7.4; 150 mM NaCl; 5 mM EDTA; 1% NP-40; 0.1% SDS) supplemented with protease inhibitors (10 μg/ml of leupeptin, aprotinin, pepstatin A, chymostatin, and 5 μg/ml of TPCK) and RNase inhibitors (100 U/ml). An aliquot of total lysate was removed from each sample for following input analysis. Equal concentration of lysate was incubated with 5 µg of FUS Ab (PA5-52610; Invitrogen, Milano, Italy) or IgG (12370; Sigma-Aldrich, Milano, Italy) under rotary agitation at 4 °C overnight. Afterwards 60 µl of slurry of Protein A/G PLUS Agarose Beads (sc-2003; Santa Cruz Biotechnology, Heidelberg, Germany) was added to each sample and incubated at 4 °C for 4 h. Then pellets were washed four times with cold TBS pH 7.6 at 3000×*g* for 5 min at 4 °C. An aliquot consisting of 10% of total beads was removed before RNA isolation from each sample for the following immunoprecipitated protein analysis by western blot. After washes, pellets of beads were resuspended in 500 µl of Trizol Reagent (Invitrogen Life Technologies, Paisley, UK) and RNAs were eluted following the manufacturer’s instructions. The immunoprecipitated RNAs with FUS and IgG control were quantized (ng/µl) using a NanoDrop 2000 spectrophotometer (Thermo, Waltham, MA, United States) and used for circCNOT6L and CNOT6L-mRNA qRT-PCR analysis, using respective primers.

### Protein extraction and western blot analysis

All murine tissues and cells (WT and *Cb1*^−/−^ testes, WT testes in vitro treated ± ACEA, WT and *Cb1*^−/−^ epididymal SPZ, total epididymal tissue from WT and *Cb1*^−/−^, *caput* and *cauda* epididymal tissue from WT, WT *caput* and *cauda* SPZ, WT *caput* SPZ in vitro treated ± ACEA, human A and B-SPZ fractions and murine E14tg2^pcDNA3_prZScan4_LNGFR^ cells transfected with *Fus* siRNA or siScr), were separately homogenized in RIPA buffer [PBS, pH 7.4, 10 mM dithiothreitol, 0.02% sodium azide, 0.1% SDS, 1% NP-40, 0.5% sodium deoxycholate, in the presence of protease inhibitors (10 μg/ml of leupeptin, aprotinin, pepstatin A, chymostatin, and 5 μg/ml of TPCK)] and sonicated three times for 30 s bursts, each at 60 mW. Proteins were separated by SDS-PAGE (8% acrylamide) and transferred to polyvinylidene difluoride membrane (GE Healthcare, Milano, Italy) at 280 mA for 2.5 h at 4 °C. The filters were treated for 3 h with blocking solution [5% nonfat milk, 0.25% Tween-20 in Tris-buffered saline (TBS, pH 7.6)] and then separately incubated overnight, at 4 °C in TBS-milk buffer (TBS pH 7.6, 3% nonfat milk) with different primary antibodies [FUS (PA5-52610), QKI (PA5-87292), RNApol2 (PA5-86234) from Invitrogen, Milano, Italy, diluted 1:500; ADAR (sc-73408) Santa Cruz Biotechnology, Heidelberg, Germany, diluted 1:500; CB1 C-terminal [32] diluted 1:500; alpha-Tubulin (sc-5286) Santa Cruz Biotechnology, Heidelberg, Germany, diluted 1:1000; MFG-E8 (sc-377356) Santa Cruz Biotechnology, Heidelberg, Germany, diluted 1:1000; Actin (E-AB-20034) Elabscience Biotechnology, Wuhan, China, diluted 1:1000]. After washing in 0.25% Tween20-TBS, filters were incubated with 1:1000 horseradish peroxidase-conjugated rabbit IgG (Dako Corp., Milano, Italy) in TBS-milk buffer and then washed again. The immune complexes were detected using the enhanced chemiluminescence-western blotting detection system [Amersham ECL western Blotting Detection Reagent (RPN2106) GE Healthcare, Milano, Italy]. Signals were quantified by densitometry analysis, adjusted relatively to Tubulin (TUB) or Actin (ACT) levels and graphed as fold change (fc) of OD values and reported as mean ± SEM. The specificity of the immunoreactions was routinely checked by omitting primary Ab (data not shown).

### Protein immunoprecipitation (IP)

For IP, 1 × 10^7^ of mice *caput* SPZ or human B-SPZ were lysed with RIPA buffer, in the presence of protease inhibitors (10 μg/ml of leupeptin, aprotinin, pepstatin A, chymostatin, and 5 μg/ml of TPCK), sonicated three times for 30 s bursts, each at 60 mW and then incubated on ice for 30 min. Lysates were centrifuged at maximum speed for 30 min 4 °C and 500 μg of supernatant proteins from each sample was incubated with 2 μg of relative Ab [FUS (PA5-52610), QKI (PA5-87292), RNApol2 (PA5-86234) from Invitrogen, Milano, Italy] or IgG (12370; Sigma-Aldrich, Milano, Italy) under rotary agitation at 4 °C overnight. Afterwards, Protein A/G PLUS Agarose Beads (sc-2003; Santa Cruz Biotechnology, Heidelberg, Germany) were added to each sample for 4 h, at 4 °C under rotary agitation. After bead incubation, samples were washed three times (3000 × g for 3 min a 4 °C) in 500 µl of cold TBS pH 7.6 and boiled in Laemmli sample buffer for 10 min to be later analyzed by SDS-PAGE.

### Functional Annotation for circRNA/miRNA and Target miRNA Interaction

Validated or predicted targets of miRNAs were retrieved by Diana TarBase 8.0 (http://www.microrna.gr/tarbase); circRNA/miRNA/Target network was built and visualized using Bisogenet plug-in of Cytoscape (https://cytoscape.org/).

### In silico analysis of circCNOT6L

RBP binding sites matching to circRNAs and flanking regions of circRNAs were predicted through CircInteractome (https://circinteractome.nia.nih.gov/index.html) (26669964). The identification of the internal ribosome entry sites (IRES) and the open reading frame (ORF) for the circRNA with protein coding potential was performed by circRNADb (version1.0.0) (http://202.195.183.4:8000/circrnadb/circRNADb.php) (27725737). Protein-RNA interaction networks from CLIP-Seq data sets were predicted through starBase v2.0 (http://starbase.sysu.edu.cn/).

### Correlation analysis

All the circCNOT6L and FUS expression values relative to *caput* and *cauda* SPZ or epididymis of WT mice have been correlated with each other. Specifically, we included in our analysis both *caput* and *cauda* SPZ or epididymal tissue, without considering the epididymal region. Data were compared using the Excel built-in distribution functions available in Microsoft Office. The value of *r* was considered to establish the test significance. The range − 1 ≤ *r* ≤ 1 established a negative or positive correlation between circCNOT6L and FUS.

### Statistical analysis

ANOVA followed by Student’s *t*-test and Duncan’s test (for multi group comparison), were used to identify groups having different mean. Differences with *P* < 0.05 were considered statistically significant. Data were expressed as the mean ± SEM from at least three independent animals for each genotype or experimental group. For qRT-PCR and western blot analyses, triplicates from each of three animals/genotype or experimental group were considered.

## Results

### CB1 involvement in circCNOT6L biogenesis in testis

**Fig. 1:**
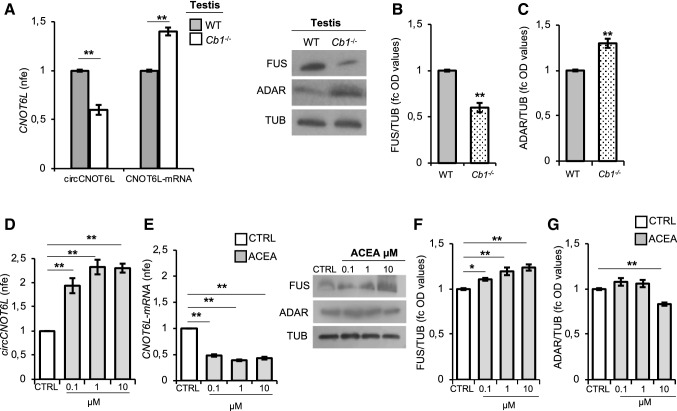
CB1 involvement in circCNOT6L biogenesis in testis. qRT-PCR detection of circCNOT6L and CNOT6L-mRNA expression levels (**A**); immunoblots and quantification of FUS (**B**) and ADAR (**C**) proteins in testis of WT and *Cb1*^*−/−*^ adult mice (*n* = 3 mice in triplicate for each group). qRT-PCR detection of circCNOT6L (**D**), CNOT6L-mRNA (**E**) expression levels and immunoblots and quantification of FUS (**F**) and ADAR (**G**) proteins in testis of WT mice in vitro treated with vehicle (CTRL) or ACEA at different concentrations: 0, 1–1–10 µM (*n* = 3 different testes in triplicate for each experimental group). In (**A**), (**D**) and (**E**), data are reported as mean value of nfe ± SEM, using *Actin* as endogenous control, while in (**B**), (**C**), (**F**) and (**G**), FUS and ADAR amount was quantified by densitometry analysis, normalized against Tubulin (TUB) signals, expressed as fc of OD values and reported as mean value ± SEM. **P < 0.01

CircCNOT6L expression has been previously correlated with sperm quality in humans [3]. Based on this, we decided to evaluate the expression levels of circCNOT6L and its mRNA counterpart (CNOT6L-mRNA) in the testis of WT and *Cb1*^*−/−*^, an animal model for poor sperm quality [19, 22] (Fig. 1A). Results showed that circCNOT6L levels were significantly lower (*P* < 0.01) in *Cb1*^*−/−*^ than in WT testis, while CNOT6L-mRNA levels were significantly higher (*P* < 0.01) in absence of CB1, suggesting that the loss of CB1 affected circ- and CNOT6L-mRNA testicular content (Fig. 1A).

In the same animals, we verified the testicular levels of FUS and ADAR proteins, considering their possible involvement in circRNA biogenesis, by western blot analysis (Fig. 1B and C). The quantitative densitometry analysis of signals showed a significantly lower FUS content (*P* < 0.01) in *Cb1*^*−/−*^ than in WT testis (Fig. 1B); conversely, ADAR levels were higher (*P* < 0.01) in *Cb1*^*−/−*^ compared to WT testis (Fig. 1C).

The possible participation of CB1 in circCNOT6L biogenesis was then verified by incubating WT testes with vehicle or ACEA—a selective cannabinoid CB1 receptor agonist—at increasing concentrations (0.1 µM, 1 µM and 10 µM). After the incubation, WT testes were processed to quantify: (i) circCNOT6L (Fig. 1D) and CNOT6L-mRNA levels (Fig. 1E) by qRT-PCR analysis; (ii) FUS (Fig. 1F) and ADAR expression (Fig. 1G) by western blot analysis.

Results showed that ACEA significantly (*P* < 0.01) increased circCNOT6L levels (Fig. 1D), while a negative effect was observed (*P* < 0.01) on CNOT6L-mRNA levels (Fig. 1E) at all doses in comparison to control group (CTRL).

ACEA treatment significantly increased FUS testicular levels at doses of 0.1 µM, 1 µM (*P* < 0.05) and 10 µM (*P* < 0.01) in comparison to CTRL group (Fig. 1F); conversely, ADAR testicular levels significantly decreased at higher dose of 10 µM (*P* < 0.01) (Fig. 1G), suggesting that circCNOT6L increase after ACEA treatment may be dependent on the modulation of FUS and ADAR enzymes.

These results clearly suggest that CB1 modulates circCNOT6L biogenesis in testis.

### Effects of *Cb1* deletion on circCNOT6L biogenesis in total epididymal SPZ

**Fig. 2:**
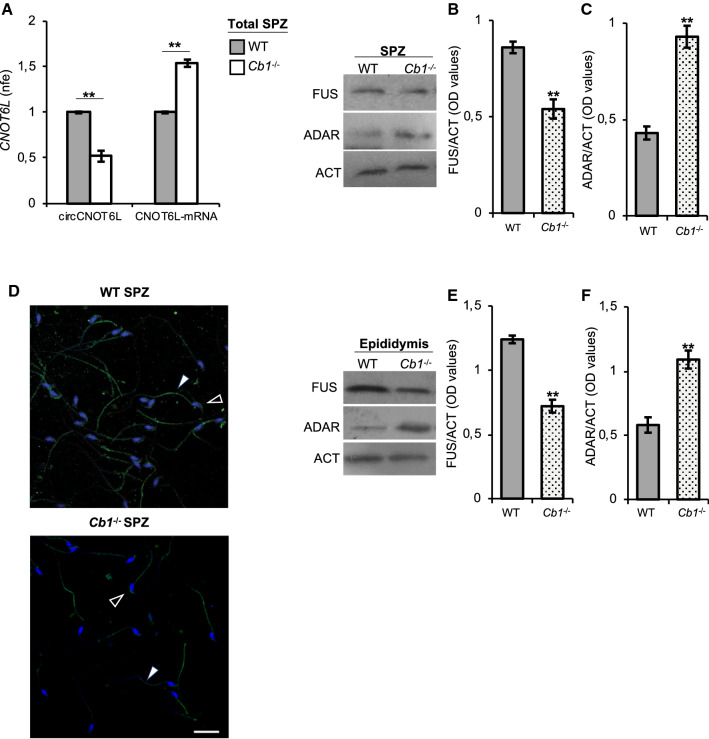
CB1 involvement in circCNOT6L biogenesis in total epididymal SPZ. qRT-PCR detection of circCNOT6L and CNOT6L-mRNA expression levels (**A**); immunoblots and quantification of FUS (**B** and **E**) and ADAR (**C** and **F**) proteins in total epididymal SPZ and/or epididymal tissue from WT and *Cb1*^*−/−*^ adult mice (*n* = 3 mice in triplicate for each group). In (**A**), the data are reported as mean value of nfe ± SEM, using *Actin* as endogenous control, while in (**B**), (**C**), (**E**) and (**F**), FUS and ADAR amount was quantified by densitometry analysis, normalized against Actin (ACT) signals, expressed as OD values and reported as mean value ± SEM. **P < 0.01. Immunofluorescence analysis of FUS protein in WT and *Cb1*^*−/−*^ SPZ (**D**). White empty arrowheads and white full arrowheads represent FUS localization (FITC-green) in sperm head and tail, respectively. Nuclei were labeled with DAPI (blue). Scale bar: 20 µM

With the aim of investigating the possibility that SPZ may be able of a backsplicing activity, total epididymal SPZ from WT and *Cb1*^*−/−*^ mice were collected and purified to evaluate: (i) circCNOT6L and CNOT6L-mRNA content by qRT-PCR analysis (Fig. 2A), (ii) FUS (Fig. 2B) and ADAR (Fig. 2C) proteins by western blot analysis, and (iii) sperm FUS localization (Fig. 2D) by immunofluorescence analysis.

*Cb1*^*−/−*^ SPZ had a significant lower and higher content of circCNOT6L and CNOT6L-mRNA, respectively, than WT SPZ (P < 0.01, Fig. 2A), thus to reflect what already found in the testis.

The quantitative densitometry analysis of signals showed a significant lower (*P* < 0.01) FUS content in *Cb1*^*−/−*^ than in WT SPZ (Fig. 2B); conversely, ADAR levels were higher (*P* < 0.01) in *Cb1*^*−/−*^ compared to WT sperm cells (Fig. 2C).

Interestingly, in WT SPZ, FUS localization was clearly defined in peri-acrosomal region and along the entire length of the tail (Fig. 2D). In addition, some WT SPZ showed FUS localization confined to the mid-piece. Conversely, in *Cb1*^*−/−*^ SPZ, although FUS localization was similar to WT, the signal strength was lower or completely absent in comparison to WT (Fig. 2D).

To assess a potential epididymal production of key modulators of backsplicing that could be shared with SPZ in transit, SPZ-deprived epididymis pieces from WT and *Cb1*^*−/−*^ mice were processed to analyze the expression levels of FUS (Fig. 2E) and ADAR (Fig. 2F) by western blot analysis. Results showed a significant decrease of FUS (Fig. 2E; *P* < 0.01) and increase of ADAR (Fig. 2F; *P* < 0.01) levels in the epididymal tissue from *Cb1*^*−/−*^ compared to WT mice.

These results suggest that SPZ contain the molecular modulators for backsplicing; their profile between WT and *Cb1*^*−/−*^ well matched with testicular and epididymal ones.

### Differential analysis of circCNOT6L in WT *caput* and *cauda* SPZ and epididymal tissue

**Fig. 3:**
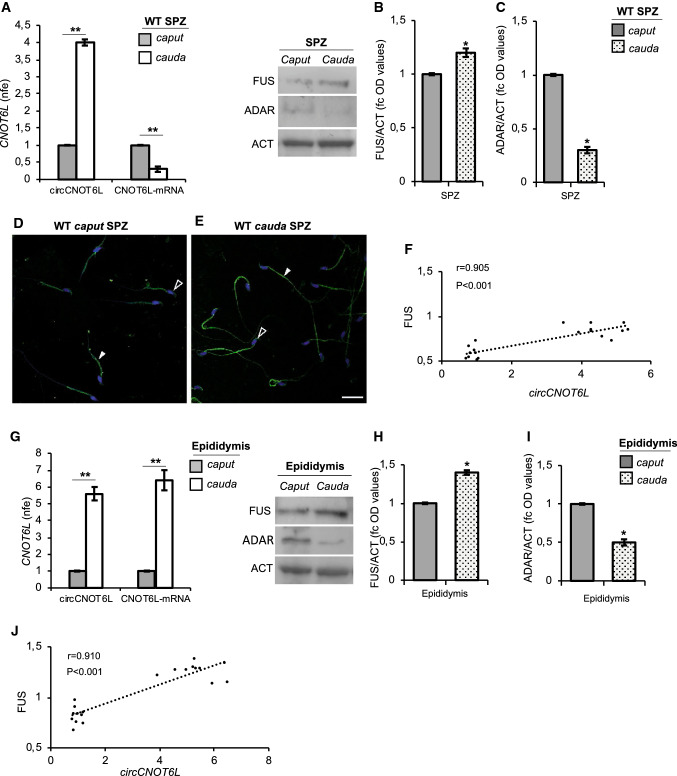
Differential analysis of circCNOT6L in WT *caput* and *cauda* SPZ and epididymal tissue. qRT-PCR detection of circCNOT6L and CNOT6L-mRNA expression levels (**A** and **G**); immunoblots and quantification of FUS (**B** and **H**) and ADAR (**C** and **I**) proteins in *caput* and *cauda* SPZ (**A**, **B** and **C**) and epididymal tissue (**G**, **H** and **I**) from WT adult mice (*n* = 3 different samples from three different mice in triplicate). In (**A**) and (**G**) data are reported as mean value of nfe ± SEM, using *Actin* as endogenous control, while in (**B**), (**C**), (**H**) and (**I**), FUS and ADAR amount was quantified by densitometry analysis, normalized against Actin (ACT) signals, expressed as fc of OD values and reported as mean value ± SEM. *P < 0.05, **P < 0.01. Immunofluorescence analysis of FUS protein in WT *caput* and *cauda* SPZ (**D** and **E**). White empty arrowheads and white full arrowheads represent FUS localization (FITC-green) in sperm head and tail, respectively. Nuclei were labeled with DAPI (blue). Scale bar: 20 µM. Correlation analysis (**F** and **J**) between circCNOT6L and FUS expression values relative to *caput* and *cauda* SPZ (**F**; *r* = 0.905, *P* < 0.001) and epididymal tissue (**J**; *r* = 0.910, *P* < 0.001) of WT mice regardless of the epididymal region

Considering that along the epididymis, SPZ mature differentially depending on the region (*caput* or *cauda*) where they stay, the sperm samples from *caput* and *cauda* epididymis of WT mice were isolated and processed to analyze: (i) circCNOT6L and CNOT6L-mRNA expression levels (Fig. 3A) by qRT-PCR analysis; (ii) FUS (Fig. 3B) and ADAR (Fig. 3C) proteins by western blot analysis, and (iii) sperm FUS localization (Fig. 3D and E) by immunofluorescence analysis.

Results showed that circCNOT6L levels significantly increased (*P* < 0.01) in SPZ from *caput* to *cauda* epididymis, while CNOT6L-mRNA levels significantly decreased (*P* < 0.01) in *cauda* SPZ in comparison to *caput* SPZ (Fig. 3A).

FUS and ADAR proteins showed very weak signals: FUS levels were significantly higher (*P* < 0.05) in *cauda* than in *caput* SPZ (Fig. 3B); conversely, ADAR levels were lower (*P* < 0.05) in *cauda* in comparison to *caput* SPZ (Fig. 3C). FUS localization in *caput* WT SPZ showed a defined regionalization in peri-acrosomal region and in the mid-piece of sperm tail (Fig. 3D). Differently, in *cauda* WT SPZ the peri-acrosomal region appeared positive for FUS signal, but more interestingly, the entire length of sperm tail became positive for FUS staining (Fig. 3E).

Additionally, *caput* and *cauda* SPZ-deprived epididymis pieces from WT mice were processed to analyze: (i) the expression levels of circCNOT6L and CNOT6L-mRNA (Fig. 3G) by qRT-PCR analysis; (ii) FUS (Fig. 3H) and ADAR (Fig. 3I) proteins by western blot analysis.

Results showed a significant increase (*P* < 0.01) of circCNOT6L from *caput* to *cauda* epididymis (Fig. 3G). Interestingly, CNOT6L-mRNA levels also significantly increased (*P* < 0.01) from *caput* to *cauda* epididymis (Fig. 3G).

A significant increase (*P* < 0.05) of FUS levels was observed in *cauda* in comparison to *caput* epididymal tissue (Fig. 3H), whereas ADAR levels significantly decreased (*P* < 0.05) in *cauda* epididymis (Fig. 3I).

We carried out a correlation analysis between circCNOT6L and FUS levels relative to WT *caput* and *cauda* SPZ (Fig. 3F) and epididymal tissue (Fig. 3J), regardless of the epididymal region. Correlation analysis showed that circCNOT6L and FUS levels were positively correlated with each other both in SPZ (Fig. 3F; *r* = 0.905, *P* < 0.001) and in epididymis (Fig. 3J; *r* = 0.910, *P* < 0.001).

These results suggest that SPZ enhance circCNOT6L biogenesis during their maturation along the epididymis.

### FUS drives CNOT6L backsplicing by recruiting RNApol2 and QKI in *caput* SPZ

**Fig. 4:**
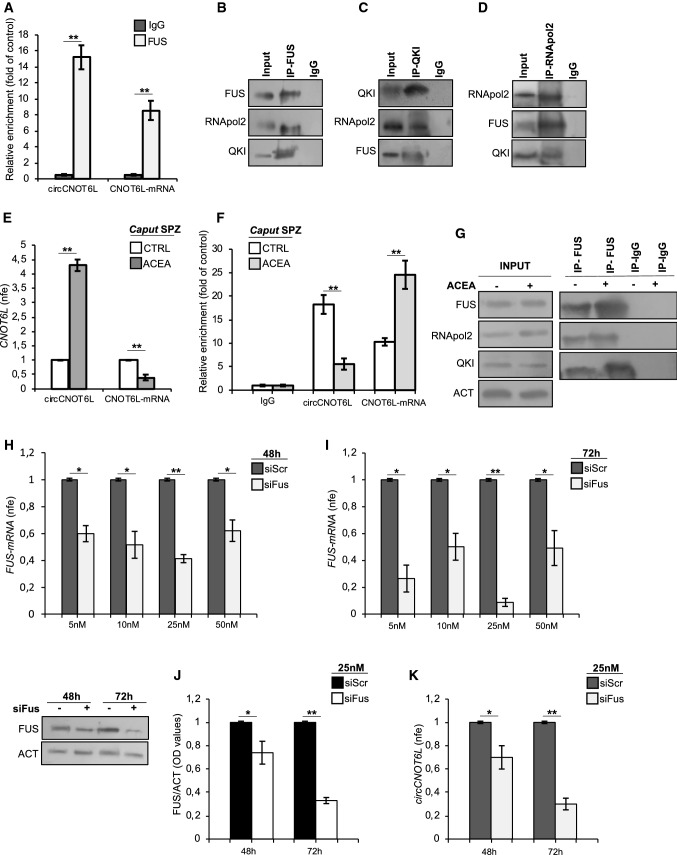
FUS drives CNOT6L backsplicing by interacting with RNApol2 and QKI in *caput* SPZ. The enrichment levels of circCNOT6L and CNOT6L-mRNA in the products of RIP assay (FUS-IP compared with IgG-IP) in WT *caput* SPZ alone (**A**) and after in vitro ACEA treatment (**F**) detected by qRT-PCR. Data are reported as mean ± SEM from three independent experiments. **P < 0.01. qRT-PCR detection of circCNOT6L and CNOT6L-mRNA expression levels (**E**) in *caput* SPZ from WT mice in vitro treated with vehicle (CTRL) or ACEA 1 µM (*n* = 3 different samples from three different animals for each experimental group in triplicate). Data are reported as mean value of nfe ± SEM, using *Actin* as endogenous control. **P < 0.01. Western blot analysis of FUS, QKI and RNApol2 in the products of IP in WT *caput* SPZ (**B**, **C** and **D**) using FUS, QKI and RNApol2 antibodies. Western blot analysis of RIP protein fraction immunoprecipitated with FUS Ab (FUS-IP) in WT caput SPZ after in vitro ACEA treatment (**G**). FUS-IP is analyzed in comparison to control IgG-IP and Input protein extracts. qRT-PCR detection of *Fus* expression levels in ESCs, cultured in RM medium, treated with siScr or siFus (5, 10, 25 and 50 nM) and harvested after 48 (**H**) and 72 h (**I**) from siRNA transfection. Data are reported as mean value of nfe ± SEM, using *Actin* as endogenous control. **P < 0.01; *P < 0.05. Western blot analysis of FUS protein (**J**) and qRT-PCR detection of circCNOT6L expression levels (**K**) in ESCs, cultured in RM medium, treated with siScr or siFus (25 nM) and harvested after 48 and 72 h from siRNA transfection. In (**J**) FUS amount was quantified by densitometry analysis, normalized against Actin (ACT) signals, and reported as mean value of fc of OD values ± SEM, while in (**K**), the data are reported as mean value of nfe ± SEM, using *Actin* as endogenous control. **P < 0.01; *P < 0.05

To evaluate the physical interaction between FUS and CNOT6L, both circRNA and mRNA, we carried out a RIP assay in WT *caput* SPZ, using FUS Ab. The results showed a 15.5- and 8.5-fold enrichment of circCNOT6L and CNOT6L-mRNA, respectively, when the anti-FUS Ab was used, relatively to the use of IgG control (Fig. 4A), reinforcing the idea of a direct interaction of FUS protein with both circCNOT6L and CNOT6L-mRNA.

Accordingly, using starBase v2.0 (http://starbase.sysu.edu.cn/), we identified some putative RBPs able to interact with circCNOT6L, focusing our attention on the ten most representative (Table 2). Interestingly, the bioinformatic analysis showed that FUS protein possessed a high number of potential target sites for the binding to circCNOT6L (Table 2).

**Table 2:** CircCNOT6L and its RBPs interactors

*RBP*	geneName	geneType	clusterNum	clipExpNum	clipIDnum
*ELAVL1*	CNOT6L	circRNA	209	7	227
*FMR1*	CNOT6L	circRNA	68	8	100
*FUS*	CNOT6L	circRNA	46	5	48
*HNRNPA1*	CNOT6L	circRNA	31	7	38
*CSTF2T*	CNOT6L	circRNA	29	7	46
*PRPF8*	CNOT6L	circRNA	26	4	35
*RBM47*	CNOT6L	circRNA	22	1	22
*U2AF1*	CNOT6L	circRNA	20	4	34
*RBM10*	CNOT6L	circRNA	9	2	9
*QKI*	CNOT6L	circRNA	3	1	3

Top 10 RBPs interacting with circCNOT6L as revealed by computational analysis of the circular sequence

Furthermore, to investigate the possibility that FUS may cooperate with other RBPs to promote circCNOT6L biogenesis, we firstly immunoprecipitated FUS from total proteins of WT *caput* SPZ (IP-FUS), followed by immunoblotting with FUS, RNApol2 and QKI antibodies (Fig. 4B). The results showed stronger FUS, RNApol2 and QKI signals in IP-FUS as compared with a significantly weaker control signals, suggesting a heterotrimeric complex formation among FUS, RNApol2 and QKI (Fig. 4B). Accordingly, the immunoprecipitation of QKI and/or RNApol2 from sperm proteins (IP-QKI and IP- RNApol2, respectively) and the immunoblotting with the same three antibodies confirmed the existence of the heterotrimeric complex in WT *caput* SPZ (Fig. 4C and D).

With the aim of strengthening the idea that SPZ may be able to circularize CNOT6L-mRNA through CB1 stimulation, SPZ collected from *caput* epididymis of WT mice were in vitro treated with 1 µM ACEA at 37 °C for 30 min. After the treatment, SPZ were processed to evaluate circCNOT6L and CNOT6L-mRNA levels by qRT-PCR analysis (Fig. 4E). A very significant increase of circCNOT6L expression was observed after CB1 stimulation by ACEA (*P* < 0.01) in comparison to CTRL (vehicle) group; conversely, CNOT6L-mRNA levels significantly decreased (*P* < 0.01) in the ACEA treated group compared to CTRL (Fig. 4E).

Therefore, CNOT6L-mRNA circularization in *caput* SPZ may be stimulated by CB1 activation and depend on both FUS/CNOT6L-mRNA and FUS-QKI-RNApol2 heterotrimeric interaction. To demonstrate this, we firstly carried out the RIP assay with FUS Ab after the in vitro treatment of *caput* SPZ with 1 µM ACEA.

Relatively to the use of IgG control, a significant increase (24.5-fold enrichment) of CNOT6L-mRNA immunoprecipitated with FUS was observed after ACEA treatment. Conversely, the results showed a reduction of FUS-circCNOT6L interaction (Fig. 4F). In addition, the western blot analysis on FUS immunoprecipitated proteins relative to RIP fractions in CTRL and ACEA groups evidenced a strong increase of FUS-QKI-RNApol2 heterotrimeric interaction after ACEA treatment (Fig. 4G). This increase was not dependent on the variations of total protein content, as confirmed by the analysis on input samples (total lysates isolated before the immunoprecipitations), suggesting an effective increased recruitment of the heterotrimeric complex on CNOT6L-mRNA (Fig. 4G).

Finally, to further prove that FUS regulates circCNOT6L biogenesis, *Fus* expression was silenced in murine ESC cells cultured in RM medium, using an RNA interference strategy. A specific pool of siRNA for *Fus* mRNA (siFus) was transfected at different doses (5 nM; 10 nM; 25 nM; 50 nM); cells were then analyzed at different time points following the transfection (48 h and 72 h). In both time points, qRT-PCR analysis showed a significant down-regulation of *Fus* expression levels (*P* < 0.01; *P* < 0.05) in comparison to the relative siScr, at all siFus doses (Fig. 4H and I). Interestingly, in both the analyzed time points the most significant reduction of *Fus* mRNA levels was observed at 25 nM of siFus (Fig. 4H and I) that was chosen for further validations. Western blot analysis confirmed a significant reduction of FUS content at 48 h (*P* < 0.05) and 72 h (*P* < 0.01) after 25 nM of siFus transfection (Fig. 4J). The expression levels of circCNOT6L after 25 nM of siFus transfection were evaluated by qRT-PCR analysis. Results showed that circCNOT6L levels were significantly reduced at 48 h (*P* < 0.05) and 72 h (*P* < 0.01) after siRNA transfection (Fig. 4K), confirming that FUS is directly involved in circCNOT6L biogenesis.

These results suggest that—under CB1 stimulation—FUS drives circCNOT6L biogenesis in *caput* SPZ by recruiting QKI and RNApol2 in a heterotrimeric complex. The direct involvement of FUS in backsplicing activity was proven by using an RNA interference strategy.

### In vitro experiment of vesicle shuttle: from epididymis to SPZ

**Fig. 5:**
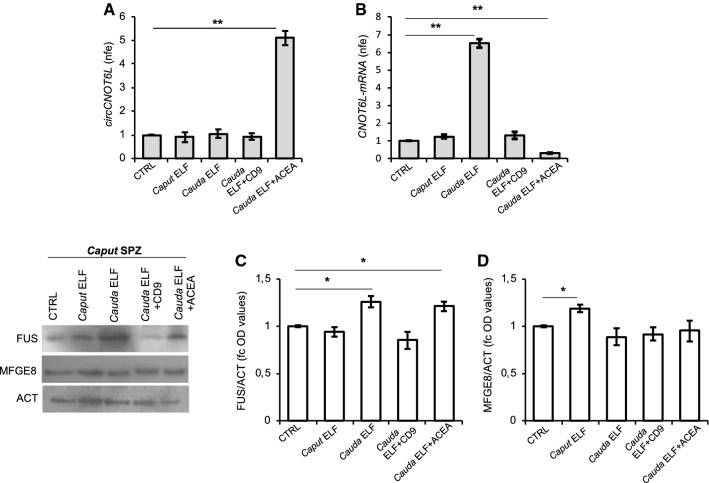
In vitro experiment of vesicle shuttle: from epididymis to SPZ. qRT-PCR detection of circCNOT6L (**A**) and CNOT6L-mRNA (**B**) expression levels in *caput* SPZ from WT mice in vitro co-incubated with: PBS (CTRL group), *Caput* ELF, *Cauda* ELF, *Cauda* ELF pre-treated with anti-CD9 Ab (*Cauda* ELF + CD9), *Cauda* ELF in presence of ACEA 1 μM (*Cauda* ELF + ACEA); (*n* = 3 different samples for each experimental group from 8 different animals in triplicate). Data are reported as mean value nfe ± SEM, using *Actin* as endogenous control. **P < 0.01. Immunoblots and quantification of FUS (**C**) and MFG-E8 (**D**) proteins in *caput* SPZ from WT mice in vitro co-incubated with: PBS (CTRL group), *Caput* ELF, *Cauda* ELF, *Cauda* ELF pre-treated with anti-CD9 Ab (*Cauda* ELF + CD9), *Cauda* ELF in presence of ACEA 1 μM (*Cauda* ELF + ACEA); (*n* = 3 different samples for each experimental group from eight different animals in triplicate). FUS and MFG-E8 amount was quantified by densitometry analysis, normalized against Actin (ACT) signals and expressed as fc of OD values. Data are reported as mean value of fc of OD values ± SEM. *P < 0.05

To assess the potential contribution of the epididymal epithelium in delivering molecules to SPZ, and, therefore, the possible sperm uptake of both CNOT6L-mRNA and FUS protein from the ELF, via epididymosomes, *caput* SPZ were in vitro incubated with *caput* or *cauda* ELF, respectively. The samples were then processed to analyze circCNOT6L and CNOT6L-mRNA expression levels by qRT-PCR analysis (Fig. 5A and B) as well as FUS levels by western blot analysis (Fig. 5C).

Results showed that CNOT6L-mRNA levels did not change in *Caput* ELF group, but significantly increased in *caput* SPZ co-incubated with *cauda* ELF (*Cauda* ELF group) in comparison to CTRL group (*P* < 0.01; Fig. 5B). Conversely, circCNOT6L levels did not change in both experimental groups (Fig. 5A).

Since the observed CNOT6L-mRNA transfer to SPZ—exclusively—via* cauda* ELF, we counteracted such an effect by an anti-CD9 masking Ab approach, previously suggested to reduce the efficacy of protein transfer from epididymosomes to SPZ [26]—prior of co-incubation with *caput* sperm. Interestingly, CNOT6L-mRNA levels returned to CTRL values (*Cauda* ELF + CD9 group, *P* < 0.01, Fig. 5B). CircCNOT6L levels still remained unchanged (Fig. 5A).

To verify a possible CB1 contribution in circRNA biogenesis, downstream the CNOT6L-mRNA transfer via vesicle shuttle from ELF, *caput* SPZ were co-incubated with *cauda* ELF in the presence of 1 µM ACEA. This dose was chosen on the basis of the results obtained in testis (Fig. 1). ACEA stimulation significantly decreased CNOT6L-mRNA levels (*P* < 0.01; Fig. 5B), increasing circCNOT6L ones (*Cauda* ELF + ACEA group, *P* < 0.01, Fig. 5A).

The same experiment was also analyzed in terms of FUS uptake from ELF by western blot analysis. FUS levels did not change when sperm was co-incubated with *caput* ELF; instead, it significantly increased after *cauda* ELF co-incubation in comparison to CTRL group (*P* < 0.05; Fig. 5C). FUS levels were similar to CTRL in *Cauda* ELF + CD9 group, whereas they significantly increased in *Cauda* ELF + ACEA group in comparison to CTRL group (*P* < 0.05; Fig. 5C).

MFG-E8 (i.e., milk fat globule-EGF factor 8) was selected as a typical exosome marker of mouse epididymosomes, with the highest abundance in the *caput* epididymosomes, useful to provide the efficacy of epididymosome cargo transfer into SPZ, considering its undetectable levels in testis [33]. Accordingly, MGF-E8 levels were significantly higher just in *Caput* ELF group in comparison to CTRL group (*P* < 0.05; Fig. 5D).

These results suggest that CNOT6L-mRNA and FUS protein are shuttled from *cauda* ELF to SPZ.

### CircCNOT6L from SPZ to oocyte and then toward the 2-cell-like state

**Fig. 6:**
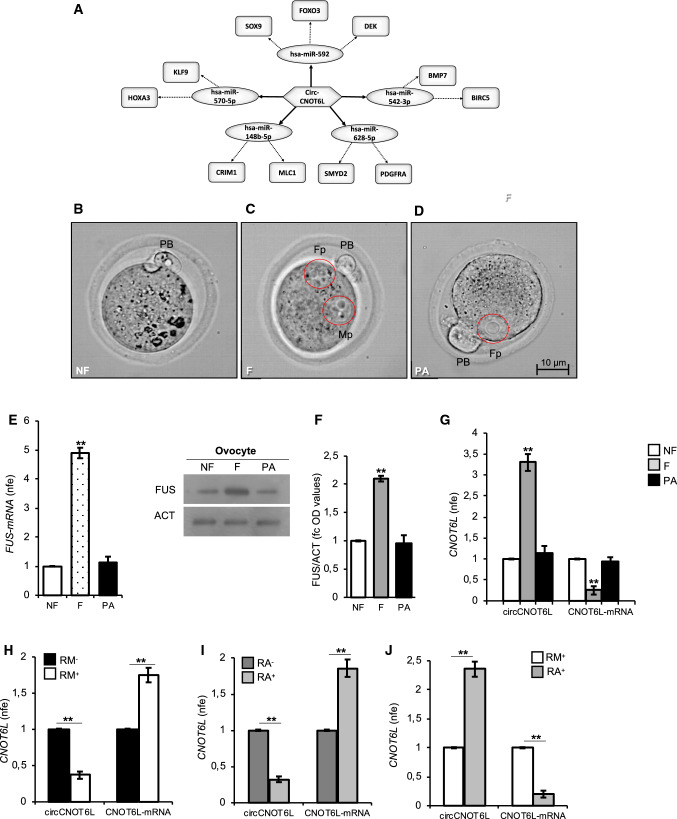
circCNOT6L from SPZ to oocyte toward the 2-cell-like state. CircCNOT6L-miRNA-mRNA network analysis was carried out by using the bioinformatic online programs (starBase, circBase, TargetScan, miRBase, Cytoscape) (**A**). Representative image of NF, F and PA (**B**, **C** and **D**). *Fp* female pronucleus, *Mp* male pronucleus, *PB* first polar globule. Scale bar: 10 µM. Expression analysis of FUS mRNA (**E**) and immunoblots and quantification of FUS protein (**F**) in NF, F and PA. *n* = 3 pools each containing 10 NF, *n* = 3 pools each containing 10 F and *n* = 3 pools each containing 10 PA were used for expression analysis. In (**E**) the data are reported as mean value of nfe ± SEM using *Actin* as endogenous control, while in (**F**), FUS amount was quantified by densitometry analysis, normalized against Actin (ACT) signals, expressed as fc of OD values and reported as mean value ± SEM. **P < 0.01. Expression analysis of circCNOT6L and CNOT6L-mRNA in NF, F, and PA (**G**) and in ESCs cultured in ES complete medium (RM) or medium supplemented with RA and magnetically separated into RM^−^ (Zscan4^−^ cells), RM^+^ (Zscan4^+^ cells), RA^−^ (Zscan4^−^ cells) and RA^+^ (Zscan4^+^ cells) (**H**, **I** and **J**, respectively) by qRT-PCR. Data are expressed as mean value of nfe ± SEM, using *Actin* as endogenous control. **P < 0.01

Considering that circRNAs are able to harbor several miRNAs [34], the construction of a circRNA-dependent network (ceRNET) is useful to shed light on predicted mRNA targets. Thus, we built a ceRNET for circCNOT6L (Fig. 6A). According to the results of bioinformatic prediction, 5 miRNAs were identified: has-miR-592, has-miR-542-3p, has-miR-628-5p, has-miR-148b-5p and has-miR-570-5p. Several downstream mRNA targets are preferentially known to be associated with embryo development, as in the case of Foxo3 and Sox9 mRNAs, respectively [35, 36]. Accordingly, Hox genes have important roles in the development of pharyngeal organs [37], Crim1 maintains retinal vascular stability [38], Smyd2 is induced during cell differentiation [39] and Bmp7—secreted by the ventral centre of *Xenopus* embryo—is involved in Spemann’s organizer [40].

Based on the FUS increase in WT *cauda* SPZ and the potential circCNOT6L involvement in embryo development, we decided to investigate a possible paternal transmission of FUS as well as CNOT6L, both circ- and mRNA, to oocytes after fertilization.

The analysis was carried out in murine NF, F—at the time in which male and female pronuclei were not fused yet—and then in PA to exclude a potential maternal contribution on FUS and CNOT6L, both circ- and mRNA, content (Fig. 6B–D).

FUS levels, both mRNA and protein, were significantly higher (*P* < 0.01) in F oocytes in comparison to NF and PA (Fig. 6E and F). Interestingly, circCNOT6L levels significantly increased (*P* < 0.01) after fertilization in F experimental group, while CNOT6L-mRNA levels were reduced (*P* < 0.01; Fig. 6G); in addition, in PA experimental group both circCNOT6L and CNOT6L-mRNA levels were comparable to their respective levels detected in NF experimental group (Fig. 6G).

With the aim of investigating the role of circCNOT6L after oocyte fertilization during the first phases of zygote development, we carried out expression analysis of CNOT6L-mRNA and circCNOT6L on RA induced transition from ESCs toward the 2-cell-like state [41, 42]. As recently reported, 2-cell-like molecular phenotype is closely associated to 2 cell preimplantation stage and is marked by Zscan4 expression [43]. Since in the absence of RA, Zscan4 expressing cells (here Zscan4^+^ cells) are a small fraction of ESC population (less than 3–5%), the generation of a modified ESC line harboring Low affinity Nerve Growth Factor Receptor gene (LNGFR) under the control of the Zscan4 promoter, was used as a strategy to efficiently collect homogeneously Zscan4^+^ cells [31].

ESCs were cultured in ES RM or ES RM supplemented with RA. Zscan4^+^ cells were isolated using a magnetically labeled anti-LNGFR Ab to separate Zscan4^+^ cells (RM^+^) from Zscan4^−^ cells (RM^−^) and Zscan4^+^ cells (RA^+^) from Zscan4^−^ cells (RA^−^).

Expression analysis carried out by qRT-PCR showed a significant reduction of circCNOT6L levels in RM^+^ compared to RM^−^ cells and conversely an increase of CNOT6L-mRNA levels in RM^+^ compared to RM^−^ cells (Fig. 6H; *P* < 0.01). Analogously, circCNOT6L levels were lower in RA^+^ than RA^−^ cells, while CNOT6L-mRNA levels were higher in RA^+^ than RA^−^ cells (Fig. 6I; *P* < 0.01).

Finally, comparative analysis between RM^+^ and RA^+^ cells showed a significant increase of circCNOT6L levels in RA^+^ compared to RM^+^ cells and conversely a reduction of CNOT6L-mRNA levels in RA^+^ compared to RM^+^ cells (Fig. 6J; *P* < 0.01).

These results suggest the paternal delivery of circCNOT6L to the oocyte. Moreover, the in vitro cell system here analyzed was useful to suggest a possible involvement of circCNOT6L in the zygote transition toward the 2-cell-like state.

### CircCNOT6L expression and biogenesis in human SPZ

**Fig. 7:**
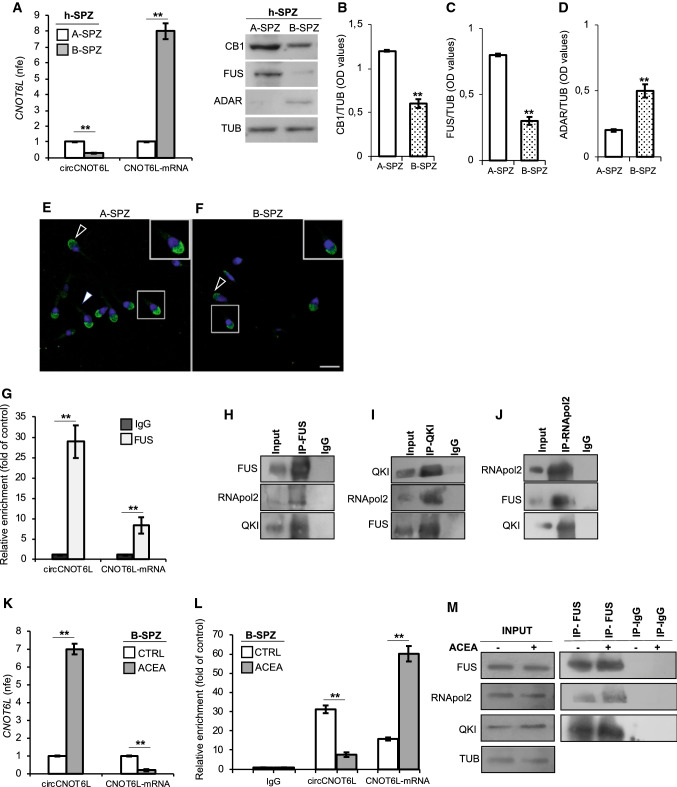
Human SPZ mimic mouse SPZ. qRT-PCR detection of circCNOT6L and CNOT6L-mRNA expression levels (**A**); immunoblots and quantification of CB1 (**B**), FUS (**C**) and ADAR (**D**) proteins in A- and B-SPZ fractions from normozoospermic volunteers (*n* = 5 different samples in triplicate). Immunofluorescence analysis of FUS protein in A- and B-SPZ (**E** and **F**). White empty arrowheads and white full arrowheads represent FUS localization (FITC-green) in sperm head and tail, respectively. Nuclei were labeled with DAPI (blue). Scale bar: 20 µM. qRT-PCR detection of circCNOT6L and CNOT6L-mRNA expression levels (**G**) in B-SPZ fractions from normozoospermic volunteers (*n* = 5) in vitro treated with vehicle (CTRL) or ACEA 1 µM (*n* = 5 different samples for each experimental group in triplicate). In (**A**) and (**G**), the data are reported as mean value of nfe ± SEM, using *Gapdh* as endogenous control. In (**B**), (**C**) and (**D**) CB1, FUS and ADAR amount was quantified by densitometry analysis, normalized against Tubulin (TUB) signals, expressed as fc of OD values and reported as mean value ± SEM. **P < 0.01. The enrichment levels of circCNOT6L and CNOT6L-mRNA in the products of RIP assay (FUS-IP compared with IgG-IP) in B-SPZ alone (**H**) and after in vitro ACEA treatment (**L**) by qRT-PCR. Data are reported as mean ± SEM from three independent experiments. **P < 0.01. Western blot analysis of FUS, QKI and RNApol2 in the products of IP in B-SPZ (**I**, **J** and **K**) using FUS, QKI and RNApol2 antibodies. Western blot analysis of RIP protein fraction immunoprecipitated with FUS Ab (FUS-IP) in B-SPZ after in vitro ACEA treatment (**M**). FUS-IP is analyzed in comparison to control IgG-IP and Input protein extracts

With the aim of characterizing circCNOT6L expression and biogenesis in human SPZ (h-SPZ), we purified good quality (A-SPZ) and poor quality (B-SPZ) SPZ from normozoospermic volunteers. In detail, circCNOT6L content significantly decreased (*P* < 0.01) in B- compared to A-SPZ; conversely, CNOT6L-mRNA levels were significantly higher (*P* < 0.01) in B- than in A-SPZ fraction (Fig. 7A).

Additionally, using the same A- and B- sperm populations, we analyzed: (i) CB1, FUS and ADAR protein levels by western blot analysis (Fig. 7B–D), and (ii) sperm FUS localization by immunofluorescence analysis (Fig. 7E and F). CB1 and FUS levels were significantly higher (*P* < 0.01) in A-SPZ than in B-SPZ (Fig. 7B and C, respectively), while ADAR content was lower (*P* < 0.01) in A-SPZ than in B-SPZ (Fig. 7D).

Immunofluorescence analysis of FUS in A-SPZ showed a clear acrosomal localization and a weak signal in the apical area of the mid-piece of sperm cells (Fig. 7E). In comparison, many B-SPZ were completely negative to FUS immunolocalization although some cells showed the same acrosomal localization of FUS, but with a weaker intensity than A-SPZ (Fig. 7F).

Additionally, the RIP assay in B-SPZ, using FUS Ab, showed a 28.8-fold enrichment of circCNOT6L and 8.2-fold enrichment of CNOT6L-mRNA relative to IgG control (Fig. 7G), confirming the direct interaction of FUS protein with both circCNOT6L and CNOT6L-mRNA in B-SPZ.

Furthermore, we immunoprecipitated FUS from total proteins of B-SPZ (IP-FUS), followed by immunoblotting with FUS, RNApol2 and QKI antibodies (Fig. 7H–J). The results showed higher FUS, RNApol2 and QKI levels in IP-FUS as compared to control, suggesting a heterotrimeric complex formation among FUS, RNApol2 and QKI (Fig. 7H). Similarly, the immunoprecipitation of QKI and/or RNApol2 from B-SPZ proteins (IP-QKI and IP- RNApol2, respectively) and the immunoblotting with the same three antibodies confirmed the heterotrimeric complex formation in these cells (Fig. 7I and J).

Thus we verified also in humans what already observed in mouse. Using the fraction of B-SPZ isolated from normozoospermic samples, we in vitro treated these with 1 µM ACEA at 37 °C for 30 min, to evaluate circ- and CNOT6L-mRNA expression (Fig. 7K) by qRT-PCR analysis. CircCNOT6L levels significantly increased in the ACEA treated group (*P* < 0.01) in comparison to CTRL group; conversely, a significant decrease of CNOT6L-mRNA levels was observed (*P* < 0.01) (Fig. 7K).

Finally, to demonstrate CB1 involvement in CNOT6L-mRNA circularization in B-SPZ by increasing FUS/CNOT6L-mRNA interaction, the RIP assay with FUS Ab was carried out in B-SPZ in vitro treated with 1 µM ACEA. As expected, relative to the use of IgG control, a significant 60-fold enrichment of CNOT6L-mRNA immunoprecipitated with FUS was observed after ACEA treatment. Instead, a 7.6-fold enrichment reduction was showed for circCNOT6L (Fig. 7L).

In addition, the western blot analysis on FUS immunoprecipitated proteins relative to RIP fractions in B-SPZ, both CTRL and ACEA-treated, evidenced a strong increase of FUS-QKI-RNApol2 heterotrimeric complex after ACEA treatment (Fig. 7M). This increase was not dependent on the variations of total protein content, as confirmed by the analysis on input samples (total lysates isolated before the immunoprecipitations), confirming an increased recruitment of heterotrimeric complex on CNOT6L-mRNA induced by CB1 activity (Fig. 7M).

These results exactly confirm in h-SPZ the molecular mechanism involved in backsplicing activity, already demonstrated in mouse SPZ.

### CircCNOT6L translatability

To verify the potential translatability of circCNOT6L, we computationally screened the circular sequence to identify interactions with RBPs, ORFs and IRES elements (Table 3). CircCNOT6L may be potentially bound by different RBPs commonly involved in stability, splicing and transport of RNAs, but not in translation. Moreover, circCNOT6L has an ORF from position 11 to 395 + 5 generating a protein sequence of 129 amino acids (aa) mostly corresponding to a leucine-rich repeat domain of CNOT6L. Hypothetical IRES elements are localized from positions 357–377 to 244–374; notwithstanding, their R-scores are too low to be considered as bona fide IRES elements.

**Table 3:** CircCNOT6L and its interactors

RBP	Binding sites	IRES (start–end)	*R* Score	Pseudoknot (Y/N)	ORF (start–end)	Protein length
AGO2	2	357–377	1.35	Y	11–1 round + 5 nt	129 aa
EIF4A3	5	244–374	1.33	N		
FMRP	2					
IGF2BP2	1					
IGF2BP3	2					
PTB	1					

A computational screening of the circular sequence to identify interaction with RBPs, ORFs and IRES elements

Accordingly, the possibility that circCNOT6L may be translated is relatively low.

## Discussion

The epigenetic signature of sperm is dynamically responsive to a wide range of environmental and lifestyle stressors [44, 45]. Sperm-derived circRNAs are included molecules. They have been recently detected in testis, seminal plasma [46–48] and SPZ [3, 27, 49].

Regulators of backsplicing are currently under investigation [50]. Three RBPs are able to bridge intronic sequences in the RNAs: QKI [51], Muscleblind (MBL) [52], and FUS [9]. A functional participation of the RNApol2 in backsplicing has also been suggested; in fact, genes able to produce circRNAs are found to be transcribed at a faster-than-average rate from RNApol2 and fruit fly mutants with a lower RNApol2 elongation rate had depleted circRNA levels [52–54]. Instead, degradation pathways and factors, as ADAR, hampering circRNA biogenesis control circRNA turnover [12]. Both FUS and ADAR have been previously analyzed in mammalian testis; the first is preferentially localized in pachytene spermatocytes [55], the second is expressed in both Sertoli and germ cells, mainly spermatogonia [56].

With the aim of investigating circRNA biogenesis in male reproductive tracts, we analyzed the expression of circCNOT6L, already detected in human SPZ of good quality [3], in *Cb1*^*−/−*^ male mice because, despite they are fertile, they produce SPZ with poor chromatin quality, DNA fragmentation, and abnormal epididymal motility acquisition [4, 19–23, 57–59]. In *Cb1*^*−/−*^ testis and SPZ, circCNOT6L content was negatively affected, as FUS levels, suggesting the possible participation of CB1 in circRNA biogenesis. To assess this hypothesis, we stimulated WT testes with ACEA, a selective cannabinoid CB1 receptor agonist [60], with a dose–response treatment chosen on the basis of pharmacological effectiveness suggested by in vitro experiments carried out in mammals [61, 62]. ACEA induced a significant increase of circCNOT6L to the detriment of its linear counterpart. In this regard, a strong competition exists between canonical splicing and backsplicing considering that both mechanisms share common canonical splice acceptors and donors [63]. However, this aspect is not without challenges due to different experimental approaches/results and therefore needs further investigation. ACEA treatment also influenced the testicular expression of FUS and ADAR proteins that increased and decreased, respectively.

The dormant transcriptional and translational state of sperm cells [64] prompted us to investigate the feasibility of an endogenous backsplicing in SPZ, during their maturation along the epididymis [65–67]. To this scope, circCNOT6L and CNOT6L-mRNA were analyzed in total SPZ collected from WT and *Cb1*^*−/−*^ mice, meanwhile FUS and ADAR expression was evaluated in both SPZ and epididymis of WT and *Cb1*^*−/−*^ mice. All the obtained patterns well matched the testicular profile, reinforcing the idea that CB1 may be involved in the molecular mechanism supporting circCNOT6L biogenesis. Furthermore, FUS localization was assessed in sperm cells by immunofluorescence analysis showing a signal in the peri-acrosomal region and along the tail of WT SPZ; *Cb1*^*−/−*^ SPZ showed a weaker signal or absence of signal.

The potential contribution of the epididymal epithelium in delivering circRNAs to SPZ was verified by collecting SPZ, separately, from *caput* and *cauda* epididymis, as well as *caput* and *cauda* epididymis fragments deprived of SPZ. CircCNOT6L and FUS increased in both *cauda* SPZ and epididymis; ADAR significantly decreased, whereas CNOT6L-mRNA decreased in *cauda* SPZ and increased in the *cauda* epididymis. The increased expression of CNOT6L-mRNA in the *cauda* epididymis may depend on the intense and continuous transcriptional activity of the epididymal epithelial cells. Accordingly, FUS intensity increased in WT SPZ from *caput* to *cauda*, extending the positivity from the mid-piece to the entire length of tail. These data would emphasize the support of the epididymal epithelial cells in circRNA biogenesis, darkening a potential ability of sperm cells to do backsplicing. However, if in *cauda* epididymis SPZ receive the correct amount of circCNOT6L from the epididymal epithelium, how to explain the increase of FUS? And why in *cauda* SPZ CNOT6L-mRNA significantly decreased? The answer may be that SPZ themselves may have an intrinsic backsplicing ability**,** supported by an epididymal-dependent uptake of both CNOT6L-mRNA and FUS protein.

With this in mind, we designed two experiments.

Firstly, we stimulated WT *caput* SPZ—producing low levels of circCNOT6L—with ACEA, and observed: (i) a significant increase of circCNOT6L; (ii) a preferential physical interaction between FUS and CNOT6L-mRNA by RIP assay, a powerful method to study the physical association between individual proteins and RNAs, in vivo [68]; (iii) a FUS-mediated recruitment of QKI and RNApol2. In this regard, several circCNOT6L-interacting RBPs were identified by a bioinformatic analysis: FUS protein possessed a high number of potential binding sites; QKI was also included, but at a lesser extent. Instead, RNApol2 was completely absent. However, considering (i) the validated role of QKI in circRNA biogenesis [50], (ii) the functional participation of the RNApol2 in controlling the balance between transcription and canonical splicing that is an important precondition for circRNA production [54], (iii) FUS ability to bind to the C-terminal domain of RNApol2 [69], we aimed to demonstrate the formation of a heterotrimeric complex among FUS, RNApol2 and QKI in SPZ.

Secondly, WT *caput* SPZ were in vitro co-incubated with *caput* or *cauda* ELF, the fluid known to vehicle epididymosomes [67, 70, 71]. The results suggested that: (i) CNOT6L-mRNA and FUS protein were shuttled from *cauda* ELF; (ii) anti-CD9 masking strategy [26, 64, 72, 73] reverted both CNOT6L-mRNA and FUS levels suggesting the epididymosome mediation; (iii) the co-incubation of *caput* SPZ with ACEA and *cauda* ELF increased circCNOT6L and decreased CNOT6L-mRNA, confirming that CB1 was a powerful signal for backsplicing reaction.

What may be the functional value of a backsplicing in sperm cells if not to provide a considerable wealth of circRNAs to the oocyte to support embryo development, as the computational analysis was suggesting? Paternal transmission of FUS as well as CNOT6L, both circ- and mRNA, to oocytes, was verified by analyzing murine NF and F, these last chosen at the time in which male and female pronuclei were not fused yet. Although FUS was already identified in murine oocytes by a proteomic approach [74], we observed its increased expression at both mRNA and protein level after fertilization likely to guarantee successful fertilization. To exclude that FUS and circCNOT6L increase in F may be just a consequence of a maternal activation, we examined PA experimental group. Parthenogenesis—a type of asexual reproduction in which the development of the female gamete takes place without fertilization—is in fact the best strategy to rule out the paternal imprint in the detected backsplicing activity. Interestingly, the content of FUS and CNOT6L, both circ- and mRNA, in PA was similar to NF, strongly supporting our hypothesis of a paternal delivery of molecules into oocyte.

The fate of circCNOT6L during the early phases of zygote development was then explored by analyzing ESC cells toward the 2-cell-like state [41, 42]. RA has been found to induce the zygotic genome activation [43], accompanied by the transition of ESCs to 2-cell-like  state, a high pluripotency state [75] and by an upsurge of Zscan4^+^ cell population [43]. Interestingly, the functional combined analysis of CNOT6L-mRNA and circCNOT6L in RM^+^ and RA^+^ cells strongly suggested that circCNOT6L may be an active modulator in zygote transition toward the 2-cell-like state.

What demonstrated in mouse SPZ was then confirmed in humans. SPZ of good quality displayed a high circCNOT6L content, a huge amount of CB1 and significant levels of FUS protein. This firing line suggested that the molecular machinery for backsplicing needs to be switched on to make SPZ suitable for fertilization. In A-SPZ, FUS was localized around the acrosome and in the apical area of the mid-piece; as expected, several B-SPZ were completely negative for FUS immunolocalization. Thus, B-SPZ—containing low levels of circCNOT6L, similarly to mouse *caput* SPZ—were treated with ACEA to induce backsplicing. CB1 stimulation increased circCNOT6L, by triggering FUS interaction with CNOT6L-mRNA and the formation of FUS-QKI-RNApol2 heterotrimeric complex, as just demonstrated in mouse.

Lastly, although it has been shown that circRNAs can be in vitro and in vivo translated to generate different protein isoforms with specific functional features [4, 76–78], we exclude for circCNOT6L this possibility, according to our computational analysis [77].

**Fig. 8:**
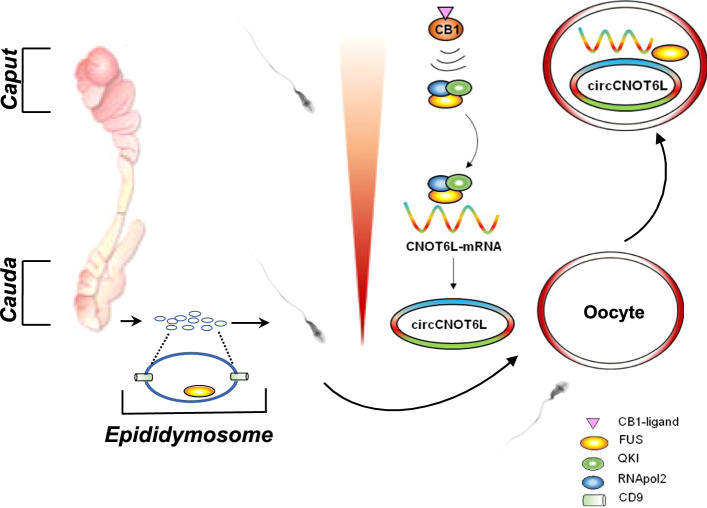
The proposed molecular mechanism at a glance. During the epididymal transit, the activation of CB1 receptor in SPZ favours the recruitment of the heterotrimeric FUS-QKI-RNApol2 complex on CNOT6L-mRNA, thus inducing the backsplicing useful for circCNOT6L biogenesis. In *cauda* epididymis, a cargo of FUS protein and CNOT6L-mRNA is transferred to SPZ via epididymosomes to support the intrinsic ability of SPZ to do backsplicing. After fertilization, the paternal content of FUS, CNOT6L-mRNA and circCNOT6L is transferred from SPZ to oocyte to sustain zygote development

In conclusion, as summarized in Fig. 8, our data suggest that along the epididymis, SPZ—not only passively receive a cargo of molecules from the epididymal epithelium—but, even in the absence of de novo gene transcription or protein translation, they are able to circularize linear transcripts through a backsplicing activity and that CB1 may be an important powerful signal.

In addition, our results support the exciting vision of the paternal delivery of molecules intended for embryo development and open new horizons for reproductive biology studies focused on the powerful role of circRNAs in this paternal inheritance of characters.

## Data Availability

The datasets in this study are available from the corresponding author upon reasonable request.
